# Acidosis and the role of nanotechnology in mitigating cancer progression

**DOI:** 10.3389/or.2026.1875516

**Published:** 2026-08-07

**Authors:** Xiaojie Liu, Rui Zhou, Ziliang Chu, Jie Liu

**Affiliations:** 1 Biochip Laboratory, Yantai Yuhuangding Hospital Affiliated to Medical College of Qingdao University, Yantai, China; 2 Ultrasound Department, Yantai Zhifu District Maternal and Child Health Hospital, Yantai, China; 3 School of Pharmacy, Yantai University, Yantai, China

**Keywords:** acidosis, metabolism reprograming, nanomaterial, pH, tumor therapy

## Abstract

Far from being a mere epiphenomenon, acidosis is a pivotal factor in the somatic evolution of cancer and its progression toward malignancy. This review provides an inclusive overview of the mechanisms by which acidosis induces and selects for malignant phenotypes. We analyze enhanced invasion and metastasis, chemoresistance, immune surveillance suppression, and metabolic reprogramming. We also briefly examine the clinical development of small-molecule and antibody-based therapies targeting pH regulation. Furthermore, this review outlines various drug delivery and therapeutic systems that respond to extracellular and/or intracellular tumor pH, highlighting the advantages of nanomaterials in precision cancer therapy. With advances in artificial intelligence, nanotechnology is poised to expedite the screening of pH-responsive systems for preclinical research, promoting the development of personalized therapies and the mitigation of adverse reactions.

## 1 Introduction

Cancer cells reside in a complex microenvironment that comprises stromal cells, immune cells, extracellular matrices, cytokines, and metabolites. The properties of this microenvironment strongly influence tumor progression (1). In 1924, Otto Warburg was the first to report that tumor cells exhibit a glycolytic phenotype even in the presence of oxygen, a phenomenon called the “Warburg Effect” (2). PET imaging has also revealed that regardless of hypoxia or metastasis, malignant tumors consume large amounts of glucose to perform efficient glycolysis and thus produce a substantial amount of lactic acid (3). Dysregulated metabolism in cancer cells, coupled with inadequate vascular perfusion, contributes to the development of extracellular acidosis (pH = 5.5–7.0). To maintain an intracellular pH compatible with their high proliferation rate, tumor cells engage in rapid buffering processes that include the metabolic consumption of non-volatile acids and the transfer of acids from the cytoplasm to organelles. Various oncogenes and tumor suppressor genes that play roles in metabolic transformation are involved in glycolysis, lactate production, and lactate/proton excretion (4). Additionally, adaptive metabolic reprogramming allows cancer cells to accumulate the biosynthetic precursors necessary for anabolic reactions, providing them with a growth advantage. This review provides a comprehensive overview of the role of acidosis in tumor progression, particularly in terms of tumor metabolism aspects, including autophagy, glutamine metabolism, mitochondrial metabolism, and fatty acid oxidation. In particular, we focus on the complex regulation of various molecular mechanisms.

Compared with traditional methods of utilizing or neutralizing acidity via small-molecule antibodies or drugs, current mainstream research is centered on the “precise manipulation of proton flow and cell fate”. Nanotechnology-based therapeutic approaches have emerged as promising strategies for improving cancer diagnosis and treatment efficiency. To overcome the limitations of passive targeting and enhance specificity, nanoparticles (NPs) can achieve active targeting via surface functionalization with ligands (e.g., antibodies, peptides, and aptamers) that recognize and bind to receptors overexpressed on cancer cells (5). Harnessing the pH gradient of tumor cells is a viable approach for improving drug delivery efficiency and therapeutic efficacy. Precise drug release is accomplished by activating the pH-responsive change in the structure of the nanomedicine upon arrival at the tumor site. This approach helps to increase drug accumulation at the tumor site, enhancing the therapeutic effect and reducing side effects. The pH-responsive design also ensures that the drugs remain stable while in the bloodstream (6, 7). Nanomaterials can enhance drug stability and bioavailability by protecting against external factors such as enzymatic hydrolysis and chemical degradation. This review summarizes cutting-edge advances in the use of nanomaterials to manipulate tumor pH gradients, including acidifying intracellular pH (pHi) and alkalizing extracellular pH (pHe). We mainly focus on delivery strategies and antitumor mechanisms and discuss how the unique pH responsiveness of nanomaterials can be used in precision cancer treatment. This review also emphasizes the limitations of some nanomaterials, such as the systemic safety of alkaline nanomaterials and whether NPs that continuously produce acid enhance immunity.

## 2 Functions of extracellular acidosis in cancer progression

### 2.1 Invasion and migration

Tumor extracellular acidosis can reduce chromosome stability and promote gene mutations, making cells more prone to malignant transformation (8). Clinical studies have shown that tumor extracellular acidosis is closely associated with poor prognosis, high metastasis rates, increased mutation rates, and resistance to radiotherapy or chemotherapy (2, 9). Human melanoma cells migrate faster at pHe 7 than at pHe 7.4. Low pH levels also induce the extracellular matrix (ECM) to promote integrin activation (10). Invadosomes, which are linked to tumor invasiveness, undergo maturation and perform functions that are stimulated by the reverse pH gradient present within the tumor (11). An acidic pericellular pH induces a redistribution of cathepsin B vesicles toward the cell periphery, potentially facilitating invasion by malignant cells (12). Acid-activated cathepsin L helps to amplify the protease cascade reaction by activating urokinase-type plasminogen activator (uPA), which cause the degradation of various ECM components, such as fibronectin, laminin, proteoglycan, and collagen (13). Cancer cells cultured at an acidic pH(e) of 6.8 secrete increased levels of matrix metalloproteinase (MMP-2 and MMP-9) and proangiogenic factors (e.g., VEGF-A), which enhance their invasive and angiogenic potential (14). Two human cell lines (A549 and HT1080) secrete higher levels of gelatinases/type IV collagenases when cultured in acidic culture media (pH 6.8) than when cultured in neutral culture media (pH 7.3), highlighting their involvement in tumor invasion and metastasis (15). Angiogenesis and lymphangiogenesis are also involved in tumor cells transmission. Transient acidosis regulates VEGF-A expression both transcriptionally and post-transcriptionally, suggesting that extracellular acidosis contributes to tumor progression (16). Esomeprazole, a proton pump inhibitor (PPI) activated by acidosis, can downregulate VEGF-C expression in acidic melanoma cells by interfering with nuclear factor–kappa B (NF-κB) activation (17). Additionally, neuron-specific acid-sensing ion channel 1 (ASIC1), which is expressed in breast cancer mediates acidosis-induced cellular signaling through phosphatidylinositol 3-kinase (PI3K)/protein kinase B (AKT). AKT then activates NF-κB and contributes to cell invasion and metastasis (18). Culturing ovarian cancer cells in an acidic medium (pH 6.6) significantly increases the production of IL-8 and promotes angiogenesis (19).

Tumor cell plasticity is influenced by the process of epithelial-mesenchymal transition (EMT). Melanoma cells exposed to acidic environment (pH 6.7) exhibit upregulation of mesenchymal markers (N-cadherin, Vimentin), and reduction of E-cadherin expression (20). An acidic extracellular tumor microenvironment (TME) provides significant protection against cell apoptosis induced by cytotoxic metabolic stresses. Low pH levels can activate mitogen-activated protein kinases (MAPK) signaling, which inhibits cell apoptosis across various cell types (21). Under acidic culture conditions, glioma cells demonstrate increased expression of stem cell markers, enhanced self-renewal, and tumor growth. *In vitro* studies suggest that raising pH can reverse the induction of HIF-2α (hypoxia-inducible factor-2α) and other tumor markers that arise from acidic stress (22, 23).

### 2.2 Chemotherapy resistance and immune escape

The acidic microenvironment outside tumor cells significantly influences their resistance to chemotherapy. An acidic pH can increase the activity of the P-glycoprotein pump, which interferes with drug distribution and promotes anti-apoptotic phenotypes. An acidic TME also impedes the flow of weakly basic chemotherapeutic drugs into cells. These drugs normally exist in a non-ionized, lipophilic form that can easily penetrate the cell membrane. However, in an acidic extracellular TME, they become heavily protonated and charged rendering them lipid insoluble and preventing them from passively diffusing across the tumor cell membrane. This phenomenon is called ion trapping. For example, the uptake of doxorubicin (DOX) and vinblastine is diminished in acidic conditions (24, 25). Furthermore, extracellular acidosis can reduce the sensitivity of tumor cells to radiotherapy. In rat breast cancer cells cultured in buffer solutions at varying pH levels, radiation sensitivity diminishes as pH decreases (26). Acidic environments can induce p53 protein expression in tumor cells and activate the p21 gene to reversibly inhibit of radiation-induced cell apoptosis (23). Furthermore, irradiation in an acidic TME prolongs the G2 phase arrest and decreases the rate of radiation-induced cell death (27).

Cancer can suppress anticancer immune responses and achieve immune escape by maintaining relatively low pH values in the TME. CD8^+^ T cells exposed to a highly acidic environment die within a few hours, and cancer cells use this “acid wall” to evade immune surveillance and attack (28). The acidic environment limits the production of important factors such as interferon-γ (IFN-γ), interleukin 10 (IL-10), and transforming growth factor-beta (TGF-β), impairing NK cell function (29), inhibiting lymphocyte proliferation, hindering dendritic cells (DCs) maturation (30), and causing T cell dysfunction (31, 32). Tumor extracellular acidification inhibits the function of cytotoxic T lymphocytes by inhibiting p38 and JNK/c-Jun activation (33). The lactate secreted by tumor cells further suppresses immune responses by increasing arginase 1 (ARG1) expression in tumor-associated macrophages and promoting IL-23/IL-17 secretion, which contributes to tumor progression (34). Exposure to H^+^ and lactate can reduce the T cell production of IL-2 and IFN-γ, as well as granzyme B and perforin, and impair the ability of monocytes to release tumor necrosis factor (35). Furthermore, lactate dehydrogenase A (LDHA) expression on cancer cells causes excess lactate accumulation to affect immune T cell cytokine production in the TME, limiting the ability of the immune system to target cancer cells (36). Latest research suggests that mitochondrial transfer is an independent pathway for cancer immune escape. Cancer cells can transfer mutant mitochondrial DNA (mtDNA) to tumor-infiltrating lymphocytes through tunneling nanotubes and extracellular vesicles. These mutant mitochondria evade degradation, ultimately leading to T cell metabolic abnormalities and senescence (37). These findings not only provide potential biomarkers for cancer immunotherapy (such as mtDNA mutations) but also provide new avenues for developing therapeutic methods targeting mitochondrial metastasis.

### 2.3 Oxidative stress

**FIGURE 1:**
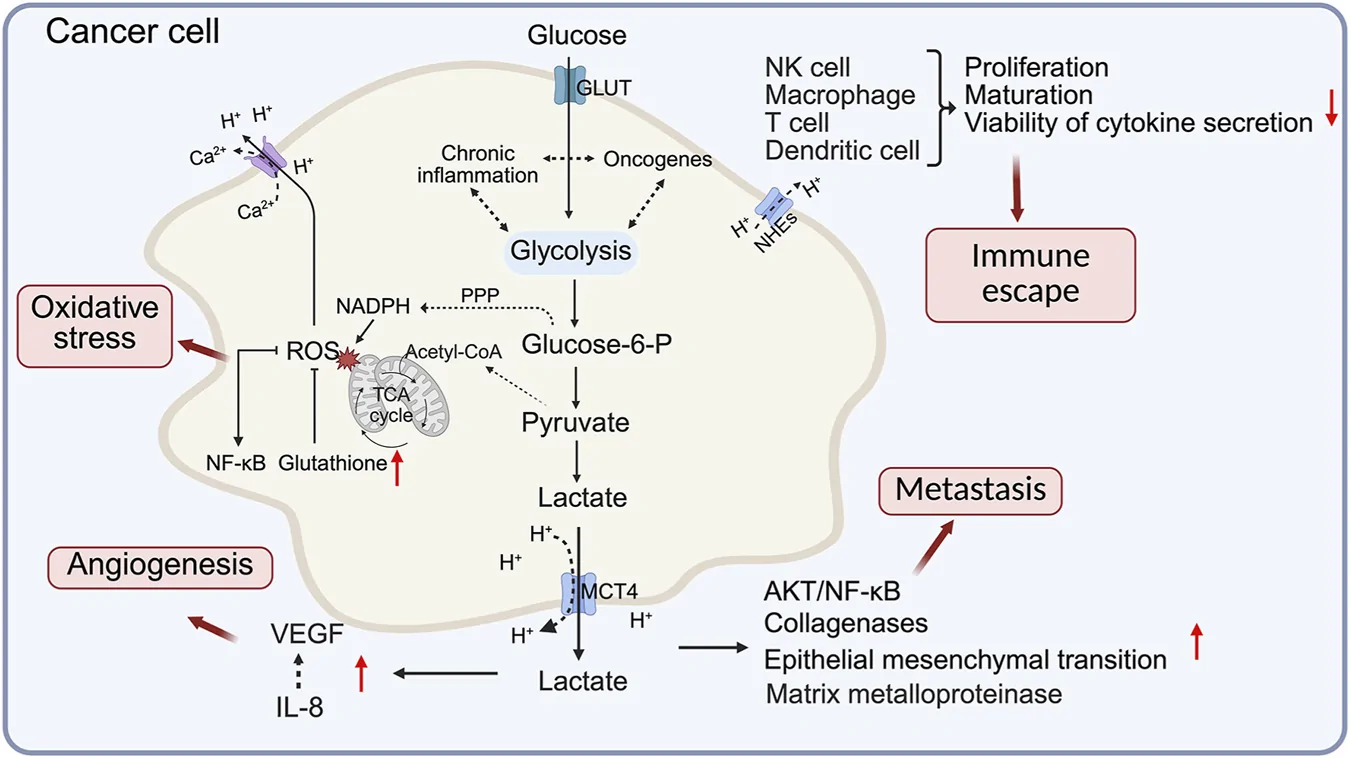
Extracellular acidosis functions in cancer. Tumor cells enhance glucose transporter protein (GLUT) expression to achieve abnormally high glucose uptake. The intracellular pH is regulated by multiple H^+^ transporter families that release protons and lactate into the extracellular environment, including Na^+^/H^+^ exchangers (NHEs), carbonic anhydrases, and monocarboxylate transporters (MCTs). MCT4 is typically upregulated in glycolysis to mediate lactate and proton efflux. Enhanced glycolysis activates NADPH oxidase and mitochondrial remodeling, promoting ROS production, which negatively regulates NF-κB activity and maintains the redox state of glutathione. The acidic environment limits cytokine production, which impairs NK cells’ function, inhibits lymphocyte proliferation, hinders dendritic cell maturation, and causes T cell dysfunction. Extracellular acidosis increases the secretion of matrix metalloproteinases, collagenases, mesenchymal markers, and proangiogenic factors, thereby enhancing their invasive and angiogenic potential. PPP, pentose phosphate pathway.

Oxidative stress is a common physiological phenomenon in the human body. The major reactive oxygen species (ROS) subtypes include superoxide radicals (O_2_·^-^), hydrogen peroxide (H_2_O_2_), and hydroxyl radicals (HO·). Under normal conditions, ROS is essential for normal physiological functions like ATP generation, various catabolic and anabolic processes, and the accompanying cellular redox cycles (38). ROS mainly originate from mitochondria and NADPH oxidase in cells. Rapid and infinite proliferation of tumor cells requires amounts of energy, leading to ROS accumulation. An acidic microenvironment further enhances ROS production from mitochondria, triggering MAPK phosphorylation. Tumor cells expressing oncogenic phosphatase of regenerating liver (PRL) protein activate a lysosome Ca^2+^ channel through ROS, causing exocytosis of the lysozyme with the plasma membrane and subsequently releasing H^+^ into the extracellular environment. This process maintains an acidic microenvironment (pHe: 6.5) (39). Tumor cells treated with an acidic medium for 6 h increase ROS level, which gradually decreases over 48–72 h due to the activity of NADPH oxidase (40). Acidosis stimulates intracellular ROS production, leading to PTEN inactivation and negatively regulating AKT-NF-κB activity, which ultimately inhibits tumor cell invasion triggered by acidosis (41, 42). However, elevated ROS levels create a selective pressure that selects tumor cells with strong antioxidant capacity and invasiveness. Colorectal cancer cells demonstrate a pro-survival mechanism that uses autophagy to boost antioxidant defense facilitating tumor survival at low extracellular pH. The GATA4-NF-κB pathway boosted antioxidant defense and enabled tumor cells to survive under low extracellular pH (43). Colorectal cancer cells continuously exposed to chronic acidic microenvironments decreased ROS levels, primarily due to elevated GSH levels (44). The redox state of glutathione and other antioxidants is determined through the continuous oxidation of ROS and the reduction of NADPH. Therefore, when tumor cells experience acidosis, insufficient NADPH hinders their antioxidant mechanisms. However, lactate plays a role in mitigating the decline in total intracellular NADPH levels during acidosis (45). Acidosis redirects glucose away from lactate production towards the oxidative branch of the pentose phosphate pathway. This is beneficial for increasing NADPH production and countering the increase in ROS present under acidosis (46). Taken together, tumor cell typically possesses a robust antioxidant capacity to eliminate ROS to maintain a relatively stable state. This stability promotes tumor cell proliferation and mutation without triggering cell death (Figure 1).

### 2.4 Metabolic reprogramming

#### 2.4.1 Glutamine (Gln) metabolism

**FIGURE 2:**
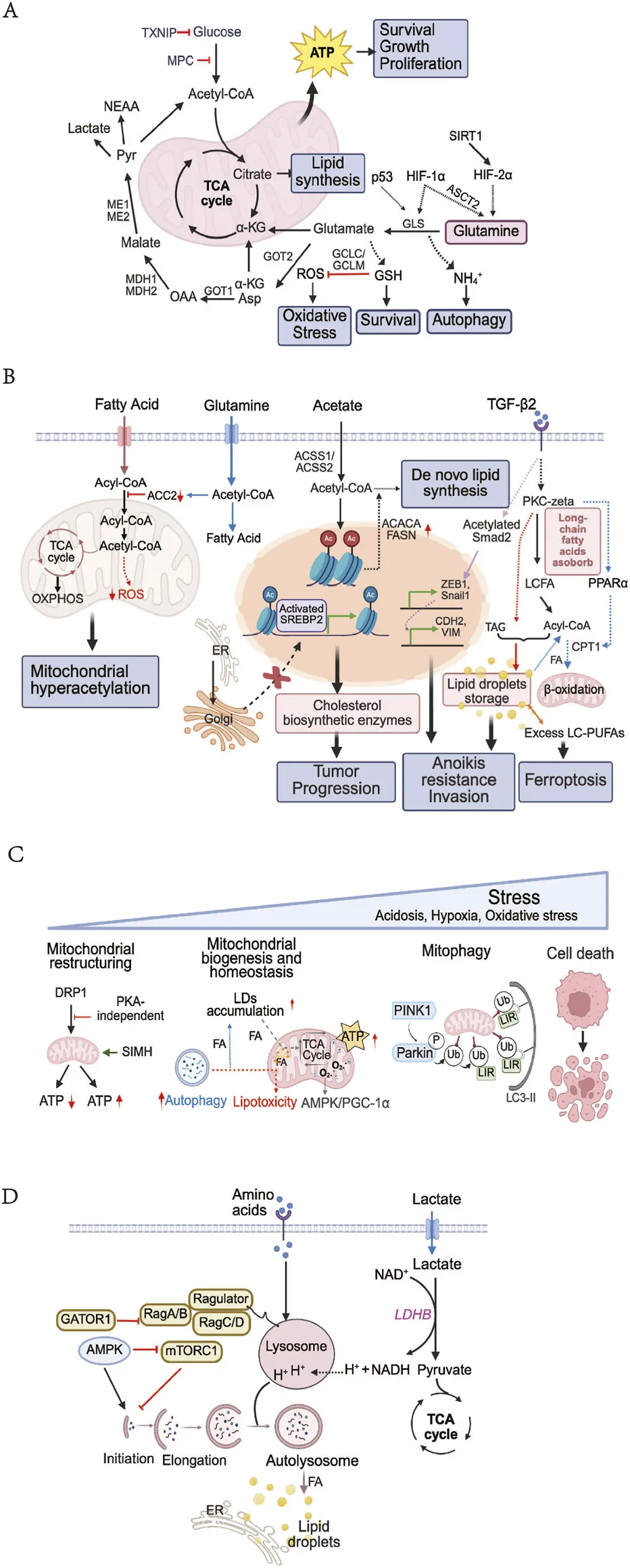
Extracellular acidosis induced metabolic reprogramming in cancer. **(A)** Tumor cells exposed to acidosis undergo glutamine metabolism reprogramming to promote growth and survival, affecting metabolic pathways such as oxidative stress, autophagy, and lipid droplet formation. NEAA, Nonessential Amino Acids. Asp, aspartate. Pyr: pyruvate. **(B)** FAs, as the center of lipid metabolism remodeling, promotes tumor cells’ survival under acidosis through mitochondrial acetylation, cholesterol synthesis, lipid droplet accumulation, and iron death. **(C)** Mitochondria regulate tumor cell response to stress (acidosis, hypoxia, oxidative stress) through restructuring, increased biogenesis, mitophagy, and cell death. Excessive FAs enter the mitochondria, where they undergo oxidation, leading to mitochondrial damage and cell apoptosis. Dysfunctional mitochondria are recognized by the PINK1/Parkin pathway for removal through autophagy. **(D)** Tumor cells adapted to acidosis demonstrate a highly active autophagic flux, which is interconnected with mitochondria, lipid droplets, and lysosomes to support their survival. FAs released by autophagy are selectively stored in lipid droplets, which are located in the endoplasmic reticulum. After being reused by tumor cells, lactate is converted into pyruvate and H^+^ through lactate dehydrogenase B (LDHB), thereby regulating the pH of lysosomal enzymes and affecting autolysosome function.

The mechanisms by which tumor cells adapt their metabolism in response to acidosis remain poorly understood. Tumor cells exposed to acidosis undergo metabolic remodeling. One key adaptation involves metabolic reprogramming toward Gln metabolism (Figure 2A). When Gln is converted to glutamate via glutaminase, its carbons enter the tricarboxylic acid (TCA) cycle as alpha-ketoglutarate, facilitated by glutamate dehydrogenase or transaminase activity. Consequently, Gln becomes one of the most critical nutrients for cell proliferation, with its carbon structure serving as a vital anaplerotic substrate that supports TCA cycle metabolism. Glutaminolysis efficiency largely relies on increased cellular uptake of Gln. Acidosis can induce the expression of thioredoxin interacting protein (TXNIP), which inhibits glucose uptake and consequently slows down glycolysis (47). Nevertheless, cancer cells depend on a high level of Gln to ensure growth in an acidic culture medium. An acidic TME further enhances NAD^+^-dependent activation of SIRT1 deacetylase, increasing iHIF-2α activity. HIF-2α promotes the expression of various transporters and enzymes that support both reductive and oxidative Gln metabolism. In contrast, HIF-1α expression is reduced, thereby further suppressing glycolysis (48). HIF-2α activation under acidic conditions drives Gln metabolism by increasing the expression of Gln transporter 2 (ASCT2) and glutaminase 1 (GLS1) in cervical cancer and colon cancer (49).

Gln decomposition supports the production of glutathione (GSH) and NADPH, which promote cell proliferation and protect cells from oxidative stress. In pancreatic ductal adenocarcinoma cells, increased expression of the glutamic-oxaloacetic transaminase enzymes (GOT1 and GOT2) enhances oxidative metabolism, particularly under low pH conditions, by activating the Gln metabolic pathway (50). Furthermore, acidosis causes a decoupling of glutaminolysis and novel GSH synthesis by repressing glutamate-cysteine ligase catalytic/modifier subunit (GCLC/GCLM) the expression (46). Gln metabolism also generates an autocrine- and paracrine-acting regulator of autophagic flux. The production of ammonia from Gln supports basal autophagy and protects cells from TNF-α induced cell death (51). Importantly, the intermediate metabolites formed during glutaminolysis help to maintain the acid-base balance. Ammonia stimulates autophagy and promotes cancer cell survival. Ammonia is also recycled to generate amino acids, including proline and aspartic acid, accelerating cancer cell proliferation. Through metabolic acidosis, the ammonium ions generated from glutaminolysis are excreted in urine, compensating for acidosis by eliminating the acids and bicarbonate ions that are transported to the blood (52). Recently, ammonia was shown to activate the dissociation of glucose-regulated N-glycosylated SREBP-cleavage-activating protein (SCAP) from insulin-inducible gene protein, thereby promoting lipogenesis by causing SREBP translocation and lipogenic gene expression (53). These studies show that targeting ammonia, the key molecular link between Gln, glucose, and lipid metabolism, is a promising strategy for treating malignancies and metabolic syndromes.

#### 2.4.2 Fatty acid oxidation (FAO)

Fatty acids (FAs) are stored in lipid droplets to maintain a dynamic balance of intracellular lipids. FAs provide energy “on demand” to promote cell membrane synthesis and energy generation. The PI3K/AKT/mTOR pathway stimulates lipogenic genes expression and enhances lipid synthesis in tumor cells (54). During periods of nutritional starvation, lipid droplets interact more with mitochondria, effectively supplying fatty acids for mitochondrial FAO (55). Under conditions of hypoxia and oxidative stress, lipid droplets formed in glial cells help limit ROS levels and inhibit polyunsaturated fatty acid (PUFA) oxidation (56). In addition, numerous FAs are transported to mitochondria, where they form excessive acylcarnitine. This accumulation may cause mitochondrial uncoupling and lipid toxicity. Thus, lipid droplets help prevent toxicity by isolating excessive FAs.

Another significant change is FAO metabolism, which enables cancer cell survival under conditions of extracellular acidosis. The shift from using glucose for energy to using FAs is a response to the acidic pH. In cancer cells from various origins, acidosis-induced TGF-β2 activation promotes PKC-zeta–mediated translocation of CD36. This translocation facilitates the uptake of FAs that are either stored as triglycerides in lipid droplets (LDs) through diacylglycerol acyltransferase 1 (DGAT1) or oxidized to generate ATP to fulfill immediate cellular needs (57). In addition, acetate activates the expression of the lipogenic genes acetyl-CoA carboxylase alpha (ACACA) and fatty acid synthase (FASN) by increasing the levels of histone (H3K9, H3K27, and H3K56) acetylation at their promoter regions, enhancing *de novo* lipid synthesis in hypoxic cancer cells (58). Generally, acetyl-CoA is oxidized and primarily used to produce ATP. However, FA-derived acetyl-CoA not only fuels the TCA cycle and supports tumor cell respiration under acidosis, but also contributes to non-enzymatic mitochondrial protein hyperacetylation. This, in turn, inhibits complex I activity and reduces ROS production (59). Extracellular acidosis activates sterol regulatory element-binding protein 2 (SREBP2) by stimulating nuclear translocation. SREBP2 inhibition suppresses the upregulation of low pH–induced genes related to cholesterol biosynthesis (60). When the buffering capacity of triglyceride storage into lipid droplets is exceeded, n-3 and n-6 PUFA peroxidation selectively induces ferroptosis in cancer cells under ambient acidosis. As a result, the peroxidability potential in acidic cancer cells increases, and long-chain n-3 and n-6 PUFA peroxidation and consecutive ferroptosis exert antitumor effects (61). Consistently, metastasis-derived cancer cells exhibit higher ferroptosis sensitivity levels and PUFA-lipid contents than cells derived from primary tumors cells (62). These studies establish dual functions of FAs in tumor progression and metastasis that may be exploitable for therapeutic development (Figure 2B).

#### 2.4.3 Mitochondria metabolism

Mitochondria, as the centers of metabolic reactions, are known as the “power stations of cells”. Although tumor cells exhibit high levels of aerobic glycolysis, mitochondria also play a critical role. In addition to producing ATP, mitochondria are involved in generating ROS and redox molecules, regulating cellular signaling, and providing intermediates as substrates for *de novo* synthesis of lipids and nonessential amino acids (63). A decrease in the mitochondrial respiratory rate is crucial for tumor cell proliferation (64). During bioenergetic crises, peroxisome proliferator–activated receptor gamma coactivator-1alpha (PGC-1α) promotes mitochondrial ATP production and energy homeostasis, enabling cells to resist necrosis and apoptosis. The PGC-1α–induced increase in mitochondrial biogenesis facilitates the development of migratory and invasive breast cancer tumor phenotypes (65). Importantly, the survival and propagation of cancer stem-like cells depend on new mitochondrial biogenesis. Similarly, enhanced mitochondrial biogenesis supports oxidative phosphorylation (OXPHOS) dependency in cancer stem cells (CSCs) (66). Prolonged exposure to acidic conditions can inhibit the glycolysis pathway and encourage FAO in T cells, enhancing mitochondria adaptability and helping “stem-like” T cells maintain their metabolic characteristics (67). Additionally, mitochondria can enable tumor cell survival under conditions of hypoxia and nutritional deprivation. Under hypoxic conditions, mitochondria preferentially activate the reductive carboxylation of Gln metabolism, producing anaplerotic intermediates for TCA; as a result, the tumor cells are heavily reliant on α-ketoglutarate to produce FAs for growth (68). Moreover, acidosis triggered by hypoxia can lead to morphological reorganization of mitochondria in mitotic cells, activating mitochondrial fusion while inhibiting fission, ultimately increasing ATP production through OXPHOS (69). Under glucose-depleted conditions, tumor cells absorb and metabolize lactate to fuel OXPHOS. Monocarboxylate transporter 1 (MCT1) is frequently harnessed by neighboring oxidative tumor cells to facilitate lactate influx for mitochondrial respiration. The rapid lactate accumulation can induce mitochondrial biogenesis, supporting the survival of lung adenocarcinoma cells (70). Notably, acute exposure to lactic acid changes tumor cell metabolism from the “Warburg” effect to an OXPHOS phenotype (71). This change is likely caused by mitochondrial pyruvate carriers that mediate mitochondrial pyruvate oxidation, functioning as a molecular switches between OXPHOS and glycolysis in various cancers (72). In fact, colorectal cancer cells adapted to acidosis become more reliant on mitochondria, which not only increase in mass but also produce more ATP. During this process, FAs derived from autophagy is channeled into LDs to prevent lipotoxicity to mitochondria. If the formation of LDs is inhibited, mitochondrial function becomes disrupted. However, this dysfunction can be restored by blocking FA transport to the mitochondria (73) (Figure 2C).

#### 2.4.4 Autophagy

Due to their rapid evolution and remarkable adaptability, tumor cells use autophagy to yield a limited supply of substrates generated from macromolecular degradation to maintain ATP production for cell survival (74). Autophagy defects can activate the DNA damage response in cancer cells, both *in vitro* and *in vivo*, increasing the likelihood of tumor development (75). Additionally, autophagy enables cancer cells to enter a dormant or diapause-like state, which contributes to therapy resistance and tumor recurrence (76). Similarly, autophagy is an important mechanism for tumor cells to maintain intracellular homeostasis and survival under acidosis. Acute low pH exposure stimulates autophagy, evidenced by an increased quantity of microtubule-associated protein light chain 3 (LC3)-positive punctate vesicles and double-membrane vacuoles, along with reduced phosphorylation of AKT and ribosomal protein S6 in breast cancer cells. Furthermore, systemically administered sodium bicarbonate increases the intratumoral pH and decreases LC3 expression in mouse tumors (77). Melanoma cells exposed to acidic culture conditions (pHe: 7.0 > pH > 6.2) promptly accumulate LC3^+^ autophagic vesicles. Bafilomycin A1–induced inhibition of lysosomal acidification further increases LC3-II accumulation, indicating that autophagic flux remains active in cells under acidic stress (78). Similarly, colon cancer cells adapted to a chronically acidic microenvironment demonstrate a highly active autophagic flux, that supports their survival. Interestingly, these cells have relatively small mitochondria, suggesting that autophagy may structurally degrade mitochondria (43). Dysfunctional mitochondria are recognized by the PINK1/Parkin pathway, as well as other regulatory molecules, allowing for their selective removal through autophagy (79). Thus, mitochondrial autophagy is essential for maintaining mitochondrial homeostasis.

Autophagy is also closely linked to lipid droplet metabolism. Mouse embryonic fibroblasts subjected to prolonged nutrient deprivation accumulate large amounts of LDs (80). In acidosis-adapted cancer cells, intracellular vacuole-like structures have been identified as LDs via electron microscopy (57). Further studies have shown that the FAs released by autophagy are selectively stored in lipid droplets by DGAT1, which is located in the endoplasmic reticulum (81). Therefore, LDs formation is a necessary outlet for autophagic degradation products. Inhibiting DGAT1 disrupts lipid homeostasis, causing excessive amounts of FAs to enter the mitochondria and undergo oxidation, producing mitochondrial damage and cell apoptosis (73). Conversely, LDs provide raw materials for the formation of autophagy membranes, thus supporting the continued process of autophagy. Liposomes can also serve as substrates for autophagy, a phenomenon referred to as lipophagy. The conserved acyl-CoA synthetase Faa1 accumulates on nucleated phagophores and locally activates FAs required for phagophore elongation and autophagy (82). Despite evolving as a pro-survival response, autophagy, especially selective types (ferritinophagy), plays a role in driving tumor cells towards ferroptosis (83). n-3 polyunsaturated FAs selectively induce ferroptosis in cancer cells under ambient acidosis (61). These findings suggest that targeting ferroptosis is an emerging antitumor approach with unique advantages against many tumor indications (Figure 2D).

### 2.5 Stemness of cancer cells

Chronic acidic microenvironment causes tumor cells to exhibit characteristics similar to cancer stem cells, including a slow proliferation rate, resistance to chemotherapy drugs, and the occurrence of epithelial-mesenchymal transformation, which leads to a dormant or diapause like state. Acidosis increases the levels and functions of HIF in glioblastoma, resulting in a marked potentiation of cancer stem cells (CSCs) self-renewal. This process occurs independently of prolyl hydroxylase domain (PHD/VHL) activity and is instead regulated by heat shock protein (HSP90) (84). Acidosis suppresses the vitamin D signaling pathway, thereby promoting the CSCs phenotype in glioma cells (85). Furthermore, it affects the stemness of colorectal carcinoma cells by modulating vitamin D receptor (VDR)–SOX2 signaling expression. Abnormal VDR expression leads to ineffective activation of vitamin D signaling, reducing its antineoplastic efficacy (86). A small subset of tumor-infiltrating lymphocytes harbors stem-like memory or precursor properties and preserves proliferative potential and persistence. Elevated extracellular acidosis impairs methionine uptake and metabolism, altering epigenetic modifications at the promoters of key T cell stemness genes, thereby maintaining their stem-like properties (67). Lactate regulates tumor dynamics by suppressing CSCs differentiation and inducing dedifferentiation into a proliferative CSCs state (87). Investigating the role of lactate and other metabolites in regulating cancer stem cells represents a promising strategy to prevent tumor relapse. As more tumor lactylation modification sites are identified, exploring the “lactate shuttle” mechanism between tumor cell and surrounding stromal cell could provide effective approaches to reverse drug resistance, enhance chemotherapy efficacy, and facilitate the development of novel therapeutics with clinical translational potential.

## 3 Treatments targeting pH

### 3.1 Small-molecule therapy

Acidosis is an important determinant of cancer progression, and tumor pH regulation is being investigated as a therapeutic target. Over the past decade, several small molecules and antibodies designed to modulate tumor cell pH have been developed and are at various stages of clinical testing. Proton pump inhibitors (PPIs) commonly used as antacids worldwide, are well tolerated and safe even when taken in high doses over extended periods to treat conditions such as Zoringer–Ellison syndrome (88). In terms of their practical application scope, various PPIs have been used for different cancers, such as liver and breast cancer. PPIs also serve as an adjuvant treatment strategy used alongside other glioma medications (89). PPIs effectively reduce tumor acidification and inhibit tumor growth in mouse models, while also countering acid-related chemical resistance (90, 91). MCT4 knockdown in combination with 3-(3-pyridinyl)-1-(4-pyridinyl)-2-propen-1-one (3-PO) can directly inhibit the glycolysis pathway, disrupting downstream lactate release and helping to overcome resistance to anti-angiogenic drugs (92).

Carbonic anhydrases (CAIX and CAXII) are another attractive therapeutic target. Chemical inhibitors of CAIX and CAXII such as SLC-0111 have been developed, and preclinical evaluations have demonstrated growth-inhibitory effects on the formation of primary tumors in various mouse models of cancer. The tyrosine kinase inhibitors imatinib and nilotinib have also been reported to reduce CAIX activity. However, further studies are needed to understand their mechanisms of action (93). CAIX-specific monoclonal antibody (girentuximab) was developed for clinical evaluation (NCT00087022) but had no clinical benefit when used as an adjuvant treatment for patients with high-risk clear cell renal cell carcinoma (ccRCC). Achieving long-term disease-free survival and overall survival represents a challenge for adjuvant ccRCC drug development (94), as well as for the application of antibody blockades to alter pH in tumor treatments.

NHE1 (Na^+^/H^+^ exchanger 1) affects tumor cell pH. Similar anticancer effects have been observed in both *in vitro* and *in vivo* experiments testing selective NHE1 inhibitors such as amiloride and caripipili (95). Although NHE1-deficient cells are phenotypically normal, their intracellular pH becomes acidic (pH = 6.8) after carcinogenic RAS expression, inducing cell death (96). Additionally, KR-33028 is a potent and selective NHE1 inhibitor. KR-33028–mediated NHE1 inhibition is highly effective in combatting the growth and spread of triple-negative breast cancer cell lines (97). Bicarbonate transporters are crucial regulators of pH levels in various types of tumors. Compounds S0859 and S3705 have been shown to reduce tumor growth in a 3D *in vitro* spheroid model and inhibit the growth of multiple breast cancer cell lines (98).

The upregulation of autophagy significantly contributes to cancer chemoresistance. Enzymes located in the autolysosomes require an acidic environment for optimum catalytic activity. LASS2 directly binds to subunit C of V-ATPase in various cancer types, indicating that LASS2 may inhibit cancer invasion and metastasis by modulating V-ATPase function (99). Additionally, diphyllin glycosides are potent inhibitors of V-ATPase, with derivatives such as compound 2l demonstrating similar inhibitory potency to that of bafilomycin A1 (100). Therefore, targeted V-ATPase inhibition may improve tumor sensitivity to chemotherapeutic drugs. Finally, combining magnetic resonance imaging (MRI) with positron emission topography scanning offers a clinical method for imaging the extracellular acidification of tumors. This method may help identify cancer patients who might respond well to pH-interfering drugs.

### 3.2 Drug therapy targeting metabolic dependencies

**FIGURE 3:**
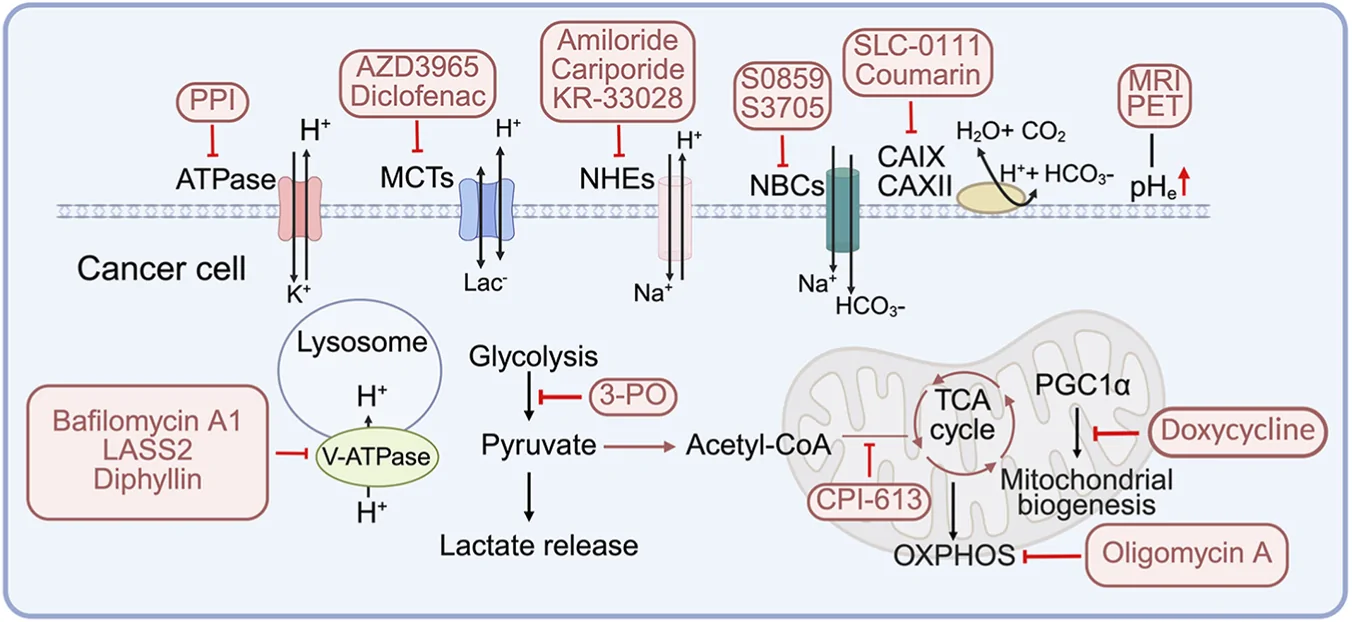
Therapeutic targeting of pH in tumors. Major pH-regulating transporters and enzymes, along with their corresponding pharmacological inhibitors, are depicted. NBCs: Na^+^/HCO_3_^−^ co-transporters. KR-33028: 4-cyano (benzo[b]thiophene-2-carbonyl) guanidine. LASS2: *homo sapiens* longevity assurance homolog 2 of yeast LAG1. Oligomycin A is an inhibitor of mitochondrial ATP-synthase.

Anticancer candidate drugs (such as CPI-613) have been shown to selectively eradicate cells adapted to acidosis by inhibiting the Krebs cycle and inducing oxidative stress (45). We agree with this hypothesis and further suggest that inducing oxidative stress in tumor cells may be a successful approach for offsetting tumor cell growth and invasion. Drug-resistant cancer cells have acquired new metabolic dependencies, making them susceptible to mitochondrial OXPHOS inhibition, which has been shown to overcome resistance to docetaxel in prostate cancer, cytarabine in acute myeloid leukemia, 5-fluorouracil in colorectal cancer, and MAPKi in melanoma (101). Therefore, multiple strategies aimed at inhibiting mitochondrial OXPHOS, such as inhibition of mitochondrial DNA replication, protein synthesis, respiratory chain complexes biogenesis, electron transfer, mitochondrial protease ClpP, and mitochondrial fatty acid transport, can enhance the antitumor effects of chemotherapy drugs. For example, doxycycline can be repurposed clinically as a mitochondrial inhibitor to target FOXM1 (a downstream target of Wnt/β-catenin signaling) and mitochondrial biogenesis in CSCs, preventing relapse by limiting tumor recurrence and distant metastasis (102) (Figure 3). Natural bioactive compounds derived from medicinal plants with anticancer and chemopreventive properties also continue to attract considerable interest (103). These plant-derived compounds have similar molecular mechanisms of action and bioavailability. Researching these natural compounds in the context of cancer metabolism may provide mechanistic insights into anticancer activity.

### 3.3 Nanomaterials targeting pH

#### 3.3.1 Elevating pHe

Using nanomaterials to neutralize acidic microenvironments is an intuitive method. For example, a novel ultrasound-responsive alkaline nanorobot (AN-DSP) has been fabricated to neutralize lactic acidosis in the TME. The nanorobots release Na_2_CO_3_ in response to ultrasound stimulation, specifically targeting and disrupting the acidic TME, improving susceptibility to anticancer drugs (i.e., DOX) and reducing drug resistance (104). In other studies, NPs have been designed to release inorganic carbonate (CaCO_3_, MnCO_3_) to neutralize acidic TME. The NPs are co-packed with chemotherapy drug/immune checkpoint antibodies to induce immunogenic cell death and improve drug penetration in tumors (105, 106) and influence the proliferation-apoptosis balance via calcium overload. Curcumin (CUR)-calcium hydroxide/oleic acid/phospholipid NPs (CUR-Ca(OH)_2_-OA/PL) are a type of hydroxide NPs that are highly internalized by tumor cells. They release CUR and Ca^2+^, intracellularly, trigger caspase 3 and caspase 9 activation, and cause apoptosis by inducing an intracellular calcium overload via a mitochondrial-mediated pathway (107). Similarly, weakly alkaline–layered double hydroxide NPs (Mg_2_Al(OH)_6_Cl) neutralize excess acid and block tumor cells autophagy when used as neoadjuvant cancer immunotherapy (108). These nanomaterials neutralize excess acid in the TME and enhance the effects of macrophages and T cells.

**FIGURE 4:**
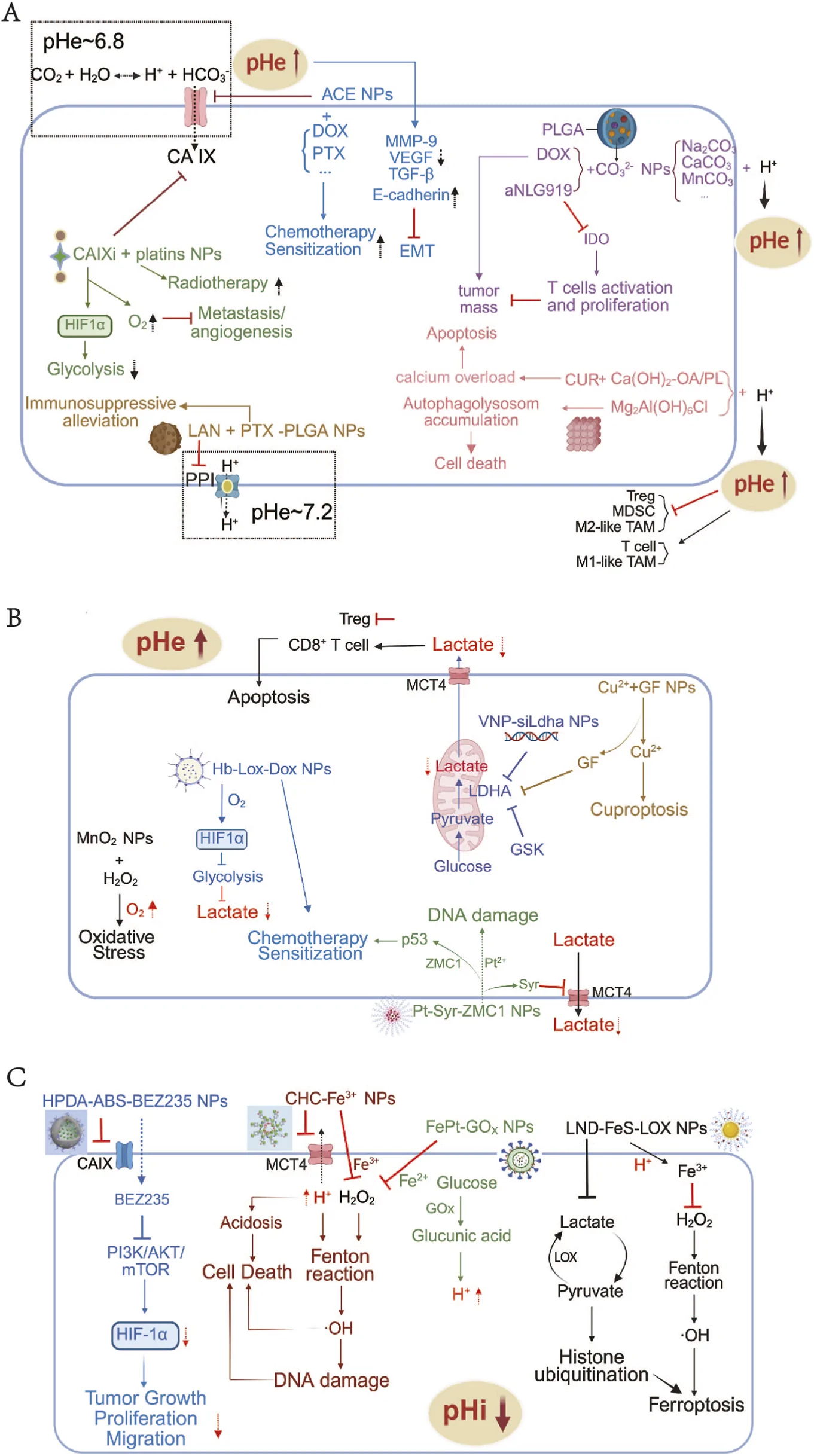
Overview of nanoparticle-mediated mechanisms regulating pHe or pHi in tumor cells. **(A)** NPs increase pHe by inhibiting CAIX or PPI. These NPs can be combined with different drugs to enhance radiotherapy effects and alleviate immunosuppression. Additionally, some NPs are designed to release inorganic carbonate or hydroxide to neutralize the acidic TME, enhancing the presence of antitumor immune cells. **(B)** Nanomaterials alkalize extracellular pH by alleviating tumor hypoxia, reducing lactate production, and inhibiting lactate transmembrane transport. This alkalization subsequently induces DNA damage and enhances antitumor efficacy of chemotherapy drugs and immunotherapy. **(C)** Lowering pHi disrupts enzyme activity, protein function, and cellular metabolism, thereby inducing tumor cell death and inhibiting proliferation. NPs reduce pHi mainly by synergizing with iron, which induces tumor cell death through the Fenton reaction or ferroptosis. GF: galloflavin. GSK: GSK2837808A.

CAIX is the main proton pump involved in pH regulation. Currently, nanomaterials loaded with CAIX inhibitors have been widely reported to achieve significant therapeutic effects. CAIX hydrolyzes external CO_2_, converting it into HCO_3_^−^ and H^+^. After crossing the plasma membrane into the cytoplasm, HCO_3_^−^ reacts with intracellular H^+^ to generate CO_2_. One study fabricated acetazolamide (ACE)-loaded pH-responsive NPs that disintegrate in acidic solutions (pH 6.8) and release ACE, inhibiting CAIX expression and increasing pHe. The combination of ACE and paclitaxel (PTX) loaded into NPs reduces tumor size and improves survival rates in mice (109). CAIX-inhibiting platinum prodrugs have also been designed, with cisplatin and oxaliplatin as the core. The platins have a strong affinity for CAIX, and the therapeutic effect of the inhibitor/platin combination is greater than that of cisplatin/oxiplatin alone (110). A CAIX-targeted self-assembling radiosensitizer (CA-Pt) enhances cisplatin penetration into deep tumor tissues and effectively inhibits CAIX, reducing HIF-1α expression and proton production. This radiosensitizer not only improves the efficiency of drug delivery but also ameliorates hypoxic and acidic TME conditions (111). Locally injecting a nanomaterial-hydrogel scaffold containing the CAIX inhibitor ACE reduces the acidity of the TME and prevents the systemic toxicity caused by ACE-induced inhibition of other carbonic anhydrases in normal tissues (112). Another innovative drug delivery system involves lansoprazole (LPZ) embedded in a polylactic acid matrix. The continuously released LPZ inhibits V-ATPase activity, blocking H^+^ excretion and neutralizing the tumor microenvironment (113). These nanomaterials achieve satisfying therapeutic effects by modifying pHe via regulation of proton pump/protein activity (Figure 4A).

Using nanomaterials to alleviate tumor hypoxia, reduce lactate production, or increase lactate utilization is another effective way to modulate the pHe. Nanoenzymes (such as ceria and manganese dioxide (MnO_2_)) are simple to prepare, are high stable, and exhibit catalytic activity. The GSH level in tumors is about four times that in normal cells, and H_2_O_2_ levels are also increased, meaning that the TME is reducible. Multifunctional bioinorganic NPs, composed of a polyelectrolyte-albumin complex and MnO_2_ generate O_2_ and increase tumor pH by reacting with the H_2_O_2_ produced by cancer cells. In a murine breast cancer model, these NPs enhanced tumor oxygenation by 45% and raised tumor pH from 6.7 to 7.2. They significantly inhibited tumor growth, induced DNA damage, and promoted cancer cell death (114). Additionally, Hb-LOX-DOX-ZIF-8 NPs were developed to target tumor acidosis and hypoxia. The NPs combined lactate oxidase (LOX) to deplete intratumoral lactate, hemoglobulin (Hb) to enhance oxygen delivery and reduce lactate production by glycolysis, and ZIF-8 encapsulation to provide acid-responsive functionality and enhance antitumor efficacy in combination with DOX chemotherapy (115). Lactate dehydrogenase (LDHA) plays a key role in lactate production. Therefore, knocking down LDHA to reduce lactate production is a feasible strategy to reduce acidity in the TME. Cationic lipid-assisted NPs that deliver siLDHA significantly reduce LDHA expression and lactate production in tumor tissues, thereby neutralizing the acidic TME (116). Conversely, Pt-Syr-ZMC1 NPs facilitate the release of syrosingopine (Syr), lowering pHe by inhibiting lactic acid secretion. Acid-activated platinum prodrug (ZMC1-Pt) responds to acidity by killing cancer cells and restoring p53 function, preventing chemoresistance (117). Recently, an emulsion gel (APEG) was developed that makes use of the water-in-oil method to deliver oxaliplatin and the LDHA inhibitor GSK. The sustained release of oxaliplatin and GSK reverses the polarization of M2-like macrophages and upregulates T cell infiltration by reducing the acidity caused by lactate (118). Programmed death-1 (PD-1) also demonstrates better therapeutic effects in tumors after acidity neutralization, demonstrating that reversing tumor acidity can enhance the efficacy of tumor immunotherapy (116). A nanoassembly constructed by integrating a lactate dehydrogenase inhibitor with an immune checkpoint inhibitor not only depleted lactate levels but also inhibited glycolysis, alleviating the immunosuppressive effects of lactate and amplifying mitochondrial cell death (i.e., cuproptosis) (119) (Figure 4B).

#### 3.3.2 Decreasing pHi for tumor treatment

Lowering pHi can disrupt cellular processes that require normal pH levels, such as enzyme activity, protein function, and cellular metabolism. This disruption can induce tumor cell death, reduce proliferation, and sensitize tumor cells to chemotherapy or other treatments. Additionally, strategies that target and modulate pHi may help overcome resistance to conventional therapies, offering a promising approach for enhancing treatment effectiveness. For example, a hydrogen sulfide (H_2_S)-driven nanomotor was created that disrupts tumor metabolic symbiosis. The nanomotor induces multiple acidifications of tumor cells and inhibits growth through the combined effects of motion and H_2_S (120). H_2_S combined with Fe^2+^ ions has been reported to inhibit catalase activity, promoting the Fenton reaction and resulting in massive ·OH production and ferroptosis via Fe^3+^. Moreover, the combination of H_2_S and a monocarboxylic acid transporter inhibitor (LND) causes intracellular acidosis by inducing the accumulation of lactate and protons (121). These nanomedicine systems, which exert precise antitumor effects by disrupting extra- and intratumoral metabolic symbiosis, represent promising active drug delivery system candidates for tumor treatment.

Chemodynamic therapy (CDT) relies on intracellular acid and hydrogen peroxide to kill tumor cells by generating hydroxyl radicals (·OH) through Fenton/Fenton-like reactions. Antitumor NPs formed by Fe^3+^ and α-cyano-4-hydroxycinnamate (CHC) inhibit MCT4 transporter function in tumor cells, thereby suppressing lactate and proton efflux, leading to intracellular acidification, and generating ·OH, which promotes tumor cell death and inhibits metastasis (122). This research provides new insights into TME regulation and CDT, offering a promising approach for treating aggressive cancers. Similarly, the biodegradable FePt system enhances the Fenton reaction via pH-sensitive liposomes and glucose oxidase (GOx). GOx promotes glucose consumption, increasing H_2_O_2_ levels and acidity in the TME. This induces ROS accumulation, leading to apoptosis and ferroptosis (123). In the field of breast cancer treatment research, a nanoplatform combining BEZ235 (PI3K/mTOR inhibitor) and ABS (CAIX inhibitor) has been developed. The platform releases BEZ235 into an acidic microenvironment to inhibit PI3K/mTOR signaling, slowing down tumor cell adaptations to hypoxia. Simultaneously, ABS enhances intracellular acidity, further inhibiting mTOR (124) (Figure 4C) (Supplementary Table S1).

### 3.4 pH-responsive nanocarriers

#### 3.4.1 Advantages of pH-responsive nanocarriers

pH-responsive nanocarriers have attracted significant attention due to their ability to facilitate targeted delivery and controlled release in response to acidic TME. Certain pH-sensitive NP carriers release therapeutic agents specifically in acidic tumor regions, while remaining stable in normal tissues. This precise targeting system maximizes therapeutic efficacy while minimizing toxicity to healthy tissues. For instance, a polymeric PROTAC (proteolysis-targeting chimeras, POLY-PROTAC) nanoplatform has been engineered for tumor-targeted degradation of the bromodomain and extraterminal (BET) protein BRD4. Dibenzocyclooctyne (DBCO)-loaded pretargeted NPs enhance intratumoral accumulation and retention of azide-modified POLY-PROTAC NPs via *in situ* click reaction. The novel POLY-PROTAC NPs respond to an acidic pH (pH < 6.8) and synergistically induce breast cancer cell apoptosis when combined with photodynamic therapy (PDT). Other stimulus-activatable chemical bonds can be used to achieve PROTAC activation as well, and other therapeutic modalities (e.g., radiotherapy) can be used in combination with the POLY-PROTAC nanoplatform (125). Another novel pH-responsive anti-polyethylene glycol (PEG) × anti-transferrin receptor (TfR) bispecific antibody system forms a complex with PEGylated nanomedicine at a physiological pH (pH = 7.4) to trigger TfR-mediated transcytosis in the brain microvascular endothelial cells. The antibody system rapidly dissociates from the PEGylated nanomedicine at acidic endosomes (pH = 6.0), efficiently crossing the BBB. This pH-responsive nanomaterial enhances the efficiency of targeted drug release, has good stability, and low potential for off-target toxicity (126).

pH-responsive nanocarriers can mediate more efficient combination therapy, enhancing tumoricidal effects. The latest pH-responsive nanomaterials combined with multimodal imaging are used to precisely identify tumor edges and perform synergistic treatment. A rapid “off-on” near-infrared fluorescent probe, NBD, with pH-activatable fluorescence has been constructed to enable near-instant and precise clinical identification of tumor margins. When the pH decreases from 7.4 to 4.0, the fluorescence quantum yield increases by a factor of 5.5. The NBD probe can identify tumor boundaries, guiding surgical tumor resection (127). A combination of multimodal near-infrared-II (NIR-II) fluorescence imaging, co-MRI, and synergistic therapy (PDT and ferroptosis) activated by an acidic pH in the tumor microenvironment has also been proposed. This system is deactivated by coordination with Fe^3+^ and activated during their release under acidic conditions. The production of hydroxyl radicals and lipid peroxides induced by ferroptosis exacerbates oxidative stress, promoting tumor cell death (128). A dual pH-responsive self-aggregating nano gold system (Au@PAH-Pt/DMMA) for the combined chemo-radiotherapy has been developed to achieve irreversible stable aggregation and pH-specific release of cisplatin prodrug. This system promotes B16 cell apoptosis as a chemotherapeutic agent and also enhances the effect of chemo-radiotherapy by promoting apoptosis as a radiosensitizer (129). Polyethylene glycol (PEG)-linked raltitrexed (RTX) has been used to modify the surface of Au NPs (∼6 nm) via lipoic acid (LA) to form a gold nanoassembly. In a mildly acid TME (pH = 6.8), the nanoassembly disassembles and splits into 6 nm Au NPs, which achieve better penetration via elevated diffusion. After disassembly, radiotherapy sensitization of the gold element and the chemotherapy effect of RTX simultaneously act on tumor cells, potentially producing better therapeutic effects (130). Researchers have also established a smart ^131^I-labeled multifunctional polyethylenimine/DOX complex with a pH-controlled cellular uptake function for enhanced single-photon emission computed tomography imaging and chemo/radioactive combination therapy. The constructed theranostic nanosystem can achieve a pH-responsive charge conversion and improve DOX cellular uptake in cancer cells under mildly acidic conditions. Complexing DOX and labeling with ^131^I enhances chemo/radioactive combination therapy (131). Moreover, the dual intervention method of targeting metabolism and immunity holds great potential for combating drug-resistant tumors. The latest research has found that the carbon-doping–engineered copper nitride enzyme (Cu_3_N-C NE) achieves steady-state reprogramming of lactate, blocking the flow of pyruvate into mitochondrial respiration, and providing an immunosuppressive TME with significant antitumor effects (132).

pH-responsive nanocarriers serve as highly efficient drug delivery systems, capable of encapsulating a diverse array of agents against a broad spectrum of cancer therapies. Nanomaterials encapsulating small-molecule drugs used in combination with chemotherapy is one of the most common approaches. For example, DOX (112, 115), paclitaxel (109), and platinum-containing compounds (117) are frequently used as NP payloads to enhance chemotherapy efficacy and modulate the TME, producing better antitumor outcomes. Nanocarriers improve drug solubility and address physicochemical and pharmacodynamic challenges associated with conventional therapies. One strategy is the co-encapsulation of multiple drugs within a single NP. However, stability issues and misaligned release kinetics can compromise treatment efficacy. The newest nanocomposites overcome drug resistance by simultaneously delivering multiple drugs with complementary mechanisms and facilitate stimulus-responsive drug release, ensuring targeted and efficient therapy. For instance, researchers have embedded chitosan hydrogels (CSHs) with mesoporous silica NPs (MSNs) to co-deliver hydrophilic 5-fluorouracil and hydrophobic everolimus (133). The amount of released drug is significantly higher at pH 5.0 than at physiological pH due to a collapse in CSH structure under acidosis. The CSHs enable sustained and precise drug release, and the MSNs exhibit a high drug loading capacity and enhanced stability. Loading different drugs separately into MSNs optimizes drug synergy. The modular design can be flexibly adapted for different drug combinations, and the pH-response characteristics apply to various solid tumors in acidic microenvironment.

#### 3.4.2 pH-responsive nanocarriers in immunotherapy

pH-responsive nanocarriers help enhance cancer immunotherapy. Acidic TME inhibits the maturation of antigen-presenting cells and the activity of lymphocytes, promoting the formation of an immune-suppressive TME. Polymer fiber-encapsulated DOX-loaded phenylboronic acid-polyethylene glycol-polycaprolactone (PBA-PEG-PCL) microparticles have been developed, as have M1 macrophages coated with PLGA NPs containing imiquimod (R837). Continuously released lansoprazole (LPZ) embedded in a polylactic acid (PLA) matrix effectively inhibits V-ATPase activity, blocking H^+^ excretion. The DOX released from the fiber matrix induces ICD and promotes DCs recruitment and maturation. R837 is preferentially engulfed by macrophages, promoting the transformation of tumor-associated macrophages (TAMs) from an anti-inflammatory phenotype (M2) to a pro-inflammatory phenotype (M1) (134). Researchers have also developed TME-responsive immunotherapeutic anti–PD-1 antibody (aPD-1) catalase-loaded calcium carbonate (CAT@CaCO_3_) NPs. These NPs consume protons in the acidic TME, normalizing the pH. The released Ca^2+^ produces a Ca^2+^ overload in the tumor cells, which triggers antitumor immune responses such as tumor antigen presentation by DCs. CAT@CaCO_3_ NPs also promote the polarization of M2 tumor-associated macrophages toward the M1 phenotype, further enhancing tumor antigen presentation. Consequently, T cell–mediated antitumor responses are activated, boosted by the aPD-1 antibody immune checkpoint blockade (135). A novel pH-responsive nanodelivery system (P-NPs) based on the amphiphilic block polymer PEO-PC7A stimulates the interferon genes (STING) agonist, activating the cGAS-STING signaling pathway and remodeling the immunosuppressive TME. In a 4T1 tumor-bearing mouse model, P-NPs inhibit tumor growth by accelerating peptide release under acidic conditions, increasing T cell cytotoxicity, and increasing IL-2 secretion (136). These studies demonstrate the advantages of pH-responsive nanomaterials in regulating the immunosuppressive TME.

The immune checkpoint blockade (ICB) has exhibited suboptimal objective response rates in some tumors, primarily due to resistance to ICB fostered by immunesuppressive TME. A dual pH-responsive nanomedicine has been successfully fabricated to co-deliver MDK-siRNA and aPD-1. The nanomedicine achieves targeted drug delivery by engaging with circulating PD-1^+^ T cells and accompanying their migration into the tumor mass. The nanomedicine promotes efficient drug release within the acidic TME, deploying aPD-1 for ICI therapy and retaining MDK-siRNA to potently suppress M2 polarization and polyamine metabolism in TAMs and myeloid-derived suppressor cells (MDSCs), with significant therapeutic efficacy in hepatocellular carcinoma (137). Partial pH-responsive nanomaterials enhance the antitumor activity of T cells by targeting immune checkpoints. Researchers have encapsulated a CAIX inhibitor (U-104) and a MRI contrast agent together in the hydrophobic poly segment core of a copolymer (OPSS-PEG-PLL-PAE) modified with AE-105 peptide to specifically target the tumoral urokinase-type plasminogen activator receptor. These NPs increase the tumor pH to ∼7.2, enhance CD8^+^ T cell infiltration, restore T cell function, and synergize with anti–PD-L1 to inhibit pancreatic ductal adenocarcinoma growth (138). Similarly, a pH-sensitive nanosized covalent-organic polymer (COP) has been developed, comprising the photosensitizer porphyrin, the pyruvate kinase inhibitor vitamin K3 (VK3), and the transcription 3 (STAT3) inhibitor WP1066. VK3 reduces intracellular oxygen consumption by inhibiting the glycolytic pathway, alleviating hypoxia. WP1066 downregulates the expression of immune checkpoint PD-L1 by inhibiting STAT3 phosphorylation, blocks the PD-1/PD-L1 immunosuppressive axis, restores the antitumor activity of cytotoxic T lymphocytes (CTLs), and ultimately achieves a synergistic antitumor effect through metabolic regulation and immune activation (139). A peptide-based nanorobot has also been designed to recognize PD-L1 and break cancer cell membranes by *in situ* by forming fibrils through a pH-responsive module (pH 6.5). The nanorobot induces immunogenic cell death and subsequent T cell infiltration and shows greater therapeutic efficacy than αPD-L1+oxaliplatin in colorectal cancer tumor–bearing mouse models (140). The above studies indicate that pH-responsive nanomaterials targeting immune checkpoints or immune-suppressive TME exhibit significant effects in terms of activating DCs, restoring T cell function, and suppressing the M2 polarization of macrophages.

## 4 Conclusion and perspectives

An acidic extracellular microenvironment is a characteristic cancer marker. Reviews have been published that summarize the effects of an acidic microenvironment on tumor progression, including immune surveillance suppression, tumor invasion promotion, autophagy, and radiotherapy and chemotherapy resistance. However, metabolic remodeling remains a crucial process in tumor cells adapted to stressful environments. Targeting metabolic characteristics is a current focus in the field of targeted therapy development. For example, mitochondria with mtDNA mutation transfer reveal a previously unknown mechanism of cancer immune evasion and may contribute to the development of future cancer immunotherapies. Lactic acid produced by tumor cells in hypoxic areas is absorbed by aerobic cells, where it serves as the main substrate for OXPHOS. Lactic acid accumulation also leads to lactylation, a novel epigenetic mechanism affecting drug resistance. Lactylation links cellular metabolism with gene expression by modifying histone and nonhistone proteins, thereby regulating chromatin accessibility, immune evasion, and DNA repair. Hence, lactic acid is no longer regarded merely as metabolic waste but as a signaling molecule involved in post-translational modification. Fang et al. (141) have proposed that lactate and lactylation exhibit significant dynamics and duality across different stages of cancer. During tumor expansion and invasion, lactate supports malignant clone selection and expansion by acidifying the microenvironment and driving immune tolerance. During cancer treatment, lactylation confers drug resistance by enhancing DNA repair and inhibiting immune responses. Therefore, the regulatory network of lactate and lactylation has become a gray area in current cancer treatment, representing an aspect of tumor survival and a key challenge in overcoming resistance and immune evasion.

The acidic microenvironments of tumors have led to the birth and development of acid-responsive intelligent nanodrug delivery systems that achieve targeted molecular dissociation, controlled drug release, and enhanced uniform distribution of drugs at the tumor site. Researchers utilize unstable chemical bonds in the acidic TME to construct pH-responsive polymers that specifically release drugs at an acidic pH (142). Common pH-sensitive bonds include hydrazone/acyl hydrazone, imine, acetal/ketal, oxime, amide, ester/orthoester, b-thiopropionatent, and borate ester bonds. NPs can be engineered to release therapeutic agents in response to specific pH changes, providing efficient and controlled drug release directly at the tumor site, and improving the internalization of drugs by tumor cells. Nanomaterials also improve drug penetration into tumors, overcoming the limitations of poor vascular permeability and heterogeneous drug distribution. Additionally, incorporating multiple drugs in a single NP system is an ideal solution for achieving combination therapies and enhancing efficacy against tumor growth and resistance. Small molecules and antibodies targeting pH modulators in tumor cells are undergoing clinical trials (94), as are several nanomedicines NP albumin–bound paclitaxel has shown good efficacy and safety in previously treated patients with advanced NSCLC (143). A clinical interventional study showed that the nanomaterial platinum acetylacetonate supported by sol-gel technology functionalized titania is beneficial in children with brain stem glioma and recurrent high-grade tumors in the central nervous system [NCT03250520]. So far, only one pH-responsive NP-based dual-activating STING agonist (ONM-501) has entered phase 1 clinical trials [NCT06022029]. ONM-501 overcomes the limitations of antigen-dependent therapy. It utilizes the acidic nature of the TME to achieve precise drug release, making it suitable for most solid tumors and lymphomas. Clinical data show that ONM-501 has high local concentration enrichment in tumors, low systemic toxicity, and a significantly better safety window than traditional immune drugs. Unfortunately, no other clinical trials of pH-responsive nanomaterials for tumor treatment have been conducted. In summary, regardless of the treatment method employed, approaches that target the acidic extracellular microenvironment are expected to be more effective than conventional chemotherapy, with different drugs specifically selected for different types of cancer.

Although pH-responsive nanocarriers have shown great potential in tumor therapy, they still face some challenges. The TME is a complex system, and pH is not the only differential factor. Therefore, to improve targeting and therapeutic efficacy, it is necessary to develop nanocarriers that respond to various stimuli. Improving the delivery efficiency of nanocarriers *in vivo* and their permeability in tumor tissues also remains a long-term challenge, and the biocompatibility and long-term safety of nanomaterials require further clinical studies. The long-term accumulation of nanomaterials *in vivo* may pose safety concerns, including chronic organ toxicity, persistent immune stimulation, and normal tissue and cellular homeostasis disruption—yet these risks have not been validated in human populations, and there remains a significant lack of long-term follow-up and multicenter controlled clinical studies. Furthermore, the *in vivo* metabolic clearance patterns of nanomaterials vary considerably depending on the type of nanomatrix (e.g., lipid nanoparticles, gold nanoparticles, and iron oxide nanoparticles), particle size, and surface modification. Establishing standardized long-term clinical observation cohorts is therefore an indispensable step in the clinical translation of nanomaterials. The future development of pH-responsive nanomaterials will focus on multi-responsive nanocarriers, which can simultaneously respond to various stimuli such as pH, redox potential, and enzymes. At present, pH-responsive nanorobots have been used in immunotherapy for mouse colorectal cancer (140). Further efforts to combine nanocarriers with artificial intelligence technology are warranted to achieve precise control of drug release and personalized treatment. In summary, the application of pH-responsive nanocarriers in tumor therapy has broad prospects. With continuous innovation and improvement, this strategy may increase the efficacy and safety of treatment options for cancer patients.
