# Supplementary material for: Acidosis and the role of nanotechnology in mitigating cancer progression
Source: Oncol Rev. 2026 Aug 7;20:1875516. doi: 10.3389/or.2026.1875516 (PMC13494587; doi:10.3389/or.2026.1875516)
Supplement: Supplementary file 1 [file Table1.PDF]

Supplementary Table 1. The Application of Representative Nanomaterials in Regulating Tumor pH

pH variation	Nanocarrier	Payload	Strategies	Anti-tumor effects	Combination therapies	Ref.
Increase in pHe; pH-responsive	Phospholipid-coating $Ca(OH)_2$ NPs	$Ca2^{+}$; CUR	Intracellular calcium overload; Lactic acidosis elimination;	Activation of caspase 3 and caspase 9; Apoptosis	/	[112]
Increase in pHe	Platelet membrane-coated ZIF8 NPs	Lactate oxidase; Hemoglobin; DOX	Lactate depletion; Hypoxia relief	Elevated ROS level; Oxygen delivery by Hb	Chemotherapy	[119]
	Acetalated dextran polymer NPs; Ace@Gel	DOX	Enhanced cytotoxicity; Metastasis inhibition	Downregulation pro-metastatic proteins; EMT blockade		[116]
Increase in pHe; pH-responsive	Polycaprolactone-b-poly NPs with a pH-responsive hydrazone bond	ACE; PTX	Tumor acidosis alleviation	Suppression of CAIX expression; Blocking CO_2 conversion to bicarbonate and a proton		[113]
Increase in pHe	PLGA NPs	DOX; $NaCO_3$; Perfluorocarbon	Neutralizing lactic acidosis; DOX resistance restoration	Na_2CO_3 neutralized lactic acidosis	Chemotherapy; Ultrasound	[108]
Increase in pHe	Allylamine hydrochloride	Albumin-MnO_2	Oxygen generation; pH increase	Enhancement of DNA double strand breaks; Downregulation of HIF and VEGF	Radiation therapy	[118]
Increase in pHe	Layered double hydroxide NPs	None (drug-free)	Excess acid neutralization; Autophagy blockade	Increased presence of macrophages and T cells	Immunotherapy	[111]
Increase in pHe; pH-responsive	$CaCO_3$@PLGA-PEG NPs	DOX; aNLG919	Immunogenic cell death; Reduction of kynurenine production by inhibiting IDO1	Increased density of tumor-infiltrating $CD8^{+}$ T cells; Depletion of Tregs	Chemo-immunotherapy	[109]
Decrease in pHi	Zwitterionic sulfobetaine methacrylate nanomotor	H_2S; α-CHCA	Induction of multiple acidosis of tumor cells	Glucose uptake increased; Inhibition of intracellular proton efflux	/	[124]

	Metal coordination polymer NPs	$Fe3^{+}$; α-cyano-4-hydroxycinnamate	Fatal acidosis; Inhibiting tumor metastasis	MCT4 inhibition; ·OH generation promotion by Fenton/Fenton-like reactions; Inhibition of lactate/H^{+} efflux; Hinderance of extracellular matrix degradation	CDT	[126]
	Hollow polydopamine	Dactolisib; 4-(2-aminoethyl) benzene sulfonamide; Chlorin e6	Mitigating hypoxic adaptation	PI3K/AKT/mTOR pathway inhibition; Suppressing HIF-1α expression; Decreasing CAIX production and angiogenesis	Phototherapy	[128]
Increase in pHe; Decrease in pHi	Polyethyleneimine-polycaprolactone (PEI-PCL) NPs	Syr; ZMC1-Pt	Inhibiting lactic acid excretion; Cell acidosis enhancement	Inhibiting the activity of MCT1 and MCT4; Wild-type p53 function restoration	Chemotherapy	[121]
pH-sensitive	Polymeric PROTAC NPs	Dibenzocyclooctyne	PROTAC-based cancer therapy	Tumour-specific protein degradation	Photodynamic therapy	[129]
	Nanoliposomes	FePt alloys; GOx	Starvation therapy	Catalysis of endogenous H_2O_2 into highly toxic ·OH by ultrasmall FePt alloys; Release of Pt(II) triggered by ROS	CDT; Chemotherapy	[127]
